# Uncommon mimics of appendicitis: giant mucocele

**Published:** 2010-10-21

**Authors:** Zahid Fatima Ezzahra, Karim Ibn Majdoub, Anoune Hicham, Abdelmalk Ousadden, Khalid Mazaz, Khalid Ait Taleb

**Affiliations:** 1 Department of General Surgery, CHU Hassan II Fes, Morocco

**Keywords:** Abdominal pain, appendicitis, mucocele, complication, malignancy, Morocco

## Abstract

Appendiceal mucocele is an infrequent but well recognized entity that can present with a variety of clinical syndromes or can be asymptomatic and discovered incidentally. A 55 years old patient was admitted in the emergency department for acute right lower quadrant pain. A diagnosis of appendicitis was made. At operation an appendiceal mucocele was found. An appendectomy was performed. The diagnosis of appendiceal mucocele is an important one in that it can be associated with malignancies and other serious gastrointestinal, ovarian, and urological complications.

## Introduction

Abdominal pain is one of the most common presenting complaints evaluated in the Emergency Department (ED). Acute right lower quadrant (RLQ) pain comprises a significant number of these presentations. A broad spectrum of common and uncommon entities may mimic acute appendicitis. Uncommon mimics include mucocele. We present a case of acute, right lower quadrant discomfort in which appendiceal mucocele was an incidental finding in the operation for acute appendicitis and discuss the pathology, associated complications, evaluation and treatment of this uncommon, but important finding. Histopathological analysis demonstrated a mucinous cystadenoma.

## Case report

**Figure 1:**
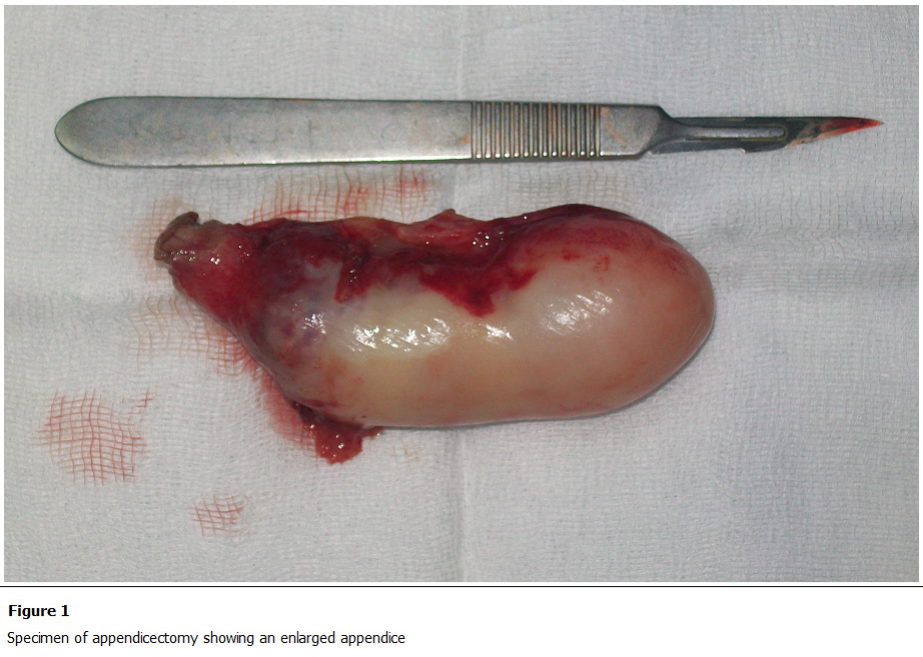
specimen of appendicectomy showing an enlarged appendice

Our patient was fiftyfive year-old male who presented with complaints of acute right lower quadrant pain, nausea and vomiting. Written permission was obtained from the patient before the publication of this case report and accompanying images. Physical examination revealed mild tenderness, right lower quadrant pain upon palpation, muscular rigidity and rebound tenderness. White blood cell count was 16000 elements/ mm3 and other laboratory values were normally. An emergency operation was performed for acute appendicitis, but an enflame mobile cystic mass with 10x7 cm in its great dimension was observed in the right iliac fossa. During the operation a diagnosis of a mucocele of the appendix was made (Figure 1). Surgical treatment included only appendectomy. Histopathological analysis demonstrated a mucinous cystadenoma.

## Discussion

Appendiceal mucocele is an uncommon abnormality, first described by Rokitansky [1]. It’s a general macroscopic description that implies a dilated appendiceal lumen caused by an abnormal accumulation of mucus. The term does not indicate etiology and includes both benign and malignant entities [1,2]. The underlying pathology can be subdivided into 4 histologic types: mucinous cystadenoma (63%), mucosal hyperplasia (25%), mucinous cystadenocarcinoma (11%) and retention cyst [3]. Mucocele can also occur due to occlusion of the lumen by endometriosis or carcinoid tumour.

The clinical presentation of appendiceal mucoceles is often nonspecific. Most mucoceles are asymptomatic, and as many as 50% are found incidentally during radiological evaluations, endoscopic procedures or surgery [1]. The most common presentation of symptomatic AM patients is acute or chronic right lower quadrant abdominal pain, as occurred with our patient. Cyclical or colicky pain can occur when AM is associated with intussusception or endometriosis. An intra-abdominal mass is palpated by the examining physician in half of cases and is also occasionally palpated by the patient. Nausea and vomiting, as well as altered bowel habits (e.g., diarrhea, constipation) are often reported, and evidence of gastrointestinal bleeding is noted if intussusception is present [3,4]. The average age at diagnosis is 50 years and, although early reports suggested that AM occurred more frequently in women than men, this predominance has been challenged in more recent studies [1].

The initial detection of the lesion may be facilitated by radiological, sonographic or endoscopic means. Ultrasound findings can be variable. Purely cystic lesions with anechoic fluid, hypoechoic masses with fine internal echoes as well as complex hyperechoic masses can be seen depending on the contents [4]. The onion skin sign is considered to be specific for mucocele of the appendix [5]. CT of the abdomen usually shows a cystic wellencapslated mass sometimes with mural calcification, in the expected location of the appendix. It may be causing extrinsic pressure on the caecal wall without any surrounding inflammatory reaction [2,6,7]. Magnetic resonance imaging has been reported useful for the evaluation of AM and also has the advantage of demonstrating any concomitant pathology [1]. In most instances, a correct pre-operative diagnosis of AM can be made with the combined use of US and CT scan [4]. On barium enema, there is usually non filling or partial filling of the appendix with contrast. The lesion may be seen as a sharply outlined sub mucosal or extrinsic mass indenting the caecum and laterally displacing it [8]. Colonoscopic findings include the “volcano sign”, the appendiceal orifice seen in the centre of a firm mound covered by normal mucosa or a yellowish, lipoma-like submucosal mass [8].

The treatment of cystic appendiceal masses is primarily surgical excision. It can either be by laparotomy or laparoscopy. Laparoscopic surgery provides the advantages of good exposure and evaluation of entire abdominal cavity, as well as more rapid recovery with avoidance of a large incision and a better cosmetic outcome. However careful handling of the specimen is recommended as spillage of the contents can lead to pseudomyxoma peritonei. This can be achieved by atraumatic handling of the appendix and use of impermeable bag for removal of the specimen. Conversion to laparotomy should be considered if the lesion is traumatically grasped or if the tumour clearly extends beyond the appendix or if there is evidence of malignancy such as peritoneal deposits [9]. Some suggest that a frozen-section examination should be performed while the abdomen is open [2].

Involvement of the caecum or adjacent organs is an indication for right hemi-colectomy and thorough exploration of the gastrointestinal tract and ovaries [10]. Patients with a simple or benign neoplastic mucocele have an excellent postoperative prognosis, with 5-year survival rates of 91%– 100%, even in cases of extension of mucus into the extra-appendiceal spaces. However, the clinical course of diffuse pseudomyxoma peritonei is insidious and unrelenting [2]. When a mucocele is present, associated neoplasms of the gastrointestinal tract, breast, ovary, and kidney must also be considered; associated colonic tumours are the most common reported.

## Conclusion

Mucocele is an uncommon pathology of the vermiform appendix that can be confused with acute appendicitis. They are difficult to diagnose and are usually discovered incidentally during operation. Appendectomy is usually sufficient; however, if the mass extends into the cecum, a right hemicolectomy may be required.

## Competing interests

The authors declare that they have no competing interests.

## Authors' contributions

Fatima ezzahra Zahid is a surgeon who drafted the manuscript and revised it critically for content. Karim Ibn Majdoub, Hicham Anoune are surgeons who are involved in literature research. Khalid Mazaz and Khalid Aït Taleb are surgeons treating of the patient and were involved in revising the draft critically for content. Abdelmalek Ousadden is a surgeon was get photographs and was involved in drafting the manuscript. All authors have given final approval of the revision to be published.
